# Outcomes of Restrictive Practice Review Meetings in an Acute Mental Health Unit: A Retrospective Before‐and‐After Study

**DOI:** 10.1111/inm.70059

**Published:** 2025-05-09

**Authors:** Esario IV Daguman, Alison Taylor, Matthew Flowers, Richard Lakeman, Marie Hutchinson

**Affiliations:** ^1^ Faculty of Health, Southern Cross University Coffs Harbour New South Wales Australia; ^2^ Integrated Mental Health, Alcohol and Other Drugs Coffs Harbour Base Hospital Coffs Harbour New South Wales Australia

**Keywords:** coercion, cognitive reflection, mental health services, physical restraint, psychiatric nursing

## Abstract

A structured process of reviewing incidents of seclusion and physical restraint supports nurses in managing the emotional and relational impact of restrictive practices. However, these reviews are not a routine feature of everyday acute care provision, and have historically been influenced by practices that reflect a managerial or disciplinary focus. A retrospective before‐and‐after study was conducted in an adult acute mental health inpatient unit in regional New South Wales, Australia. The goal was to determine the impact of strengths‐based restrictive practice review meetings from January 2019 to March 2020, with an equivalent timeframe before its implementation (October 2017–December 2018). The events of seclusion, physical restraint, and Code Blacks (emergency codes requiring security personnel in response to personal safety threats) were compared before and after the implementation of review meetings, with covariate balancing applied to enhance comparability. The reduction in the rate of seclusion was statistically significant post‐implementation (incidence rate ratios [IRR] = 0.37, 95% CI [0.24, 0.57], *p* < 0.001). At the same time, physical restraint showed a statistically insignificant reduction (IRR = 0.76, 95% CI [0.53, 1.09], *p* = 0.14). Code Black incidents remained stable (IRR = 1.02, 95% CI [0.70, 1.49], *p* = 0.91). These mixed results indicate that strengths‐based reviews play a role in reducing coercion, although additional strategies may be needed to achieve significant reductions in restrictive practice outcomes. Intervention development requires broader service‐level changes, with open and robust evaluations that enhance accountability and capture key influencing factors.

## 1 Introduction

Engaging in a reflective process that provides opportunities to learn from, receive support during, and be changed by, the everyday practice of acute care provision is essential for mental health nurses. These opportunities can support reflection on technical and relational competencies, especially after deploying restrictive practices (Goulet and Larue 2016). However, this form of reflection is typically employed in response to physical aggression or violence, is used inconsistently, and has varying levels of acceptance among staff and services (Mangaoil et al. 2023). This current state is unhelpful, as exposure to restrictive practices can be distressing for nurses, negatively impact their well‐being, and decrease the quality of care they provide (Lamoureux et al. 2024). Nurses need the opportunity not only to recover from any threat or physical injury, but also to address the moral distress of bearing witness to inadequate treatment and service users suffering (Jansen et al. 2022).

In acute care mental health settings, restrictive practices, such as seclusion and restraint, are legally regulated measures that limit a person's privacy and freedom (De Cuyper et al. 2023). Service users and their supporters consider seclusion and restraint as dehumanising, traumatic, anti‐recovery, and isolating (Brophy et al. 2016). In response, it is a policy priority for mental health services worldwide to minimise and, if possible, eliminate restrictive practice use, alongside reviewing and reporting these incidents to identify areas for improvement (e.g., NSW Health 2017). Despite this direction, reductions in restrictive practice use remain unrealised, with service‐level factors contributing to its variation. In Australia, seclusion continues to be used, while physical restraint remains at 10 events per 1,000 bed days from 2021–2022 to 2022–2023 (AIHW 2024).

Restrictive practice reviews offer an avenue for open discussion and reflection on care improvement (Berring et al. 2024). Nurses, the largest professional group in mental health services worldwide (Joseph et al. 2022), see these reviews as stress‐reducing, restorative for therapeutic and collegial relationships (Gustafsson and Salzmann‐Erikson 2016), heightening trauma awareness, and helping them respond emotionally (Goulet et al. 2018). Programmes with restrictive practice reviews have been associated with reducing both the frequency and duration of seclusion and restraint (Ashcraft and Anthony 2008; Fisher 2003; Goulet et al. 2018; Riahi et al. 2016).

Although there is evidence for the value and effectiveness of restrictive practice reviews, notable gaps remain, particularly in their scope and focus. Despite the adoption of strengths‐based approaches, it is not uncommon for programmes aimed at reducing restrictive practices in adult inpatient care to focus on tracking and communicating failed interventions over successful de‐escalations (Baker et al. 2021). Reviews solely involving care providers tend to centre on discussing events of seclusion and restraint, their triggers, and any possible non‐coercive alternatives (Hammervold et al. 2019). While this emphasis supports objectivity, it is unclear whether these reviews explicitly recognise the efforts of mental health nurses to be affirmed for what they did well.

Beyond content limitations, programmes incorporating restrictive practice reviews are sparse in quantity and evaluation quality. In an overview of systematic reviews, only three out of 23 initiatives to reduce restrictive practice use included an element of restrictive practice review (Daguman et al. 2024). Where reviews were included, evaluations tended to have low to moderate methodological quality and limited evidence certainty (Allen et al. 2018; Gaynes et al. 2017). Controlling for confounds has also not been commonly applied, which raises concerns about biased findings and limits the reliability of conclusions made about the interventions. Code Blacks—emergencies requiring the presence and support of security personnel in response to personal threats—have not been considered in evaluations, despite their role in supporting staff safety and creating power imbalances for service users in acute units (Muir‐Cochrane et al. 2020).

Supporting mental health nurses' moral and esteem needs is essential in change initiatives, as they are said to face blame both for using restrictive practices and for the consequences of not using them (Muir‐Cochrane et al. 2018). This blame can erode trust and respect among colleagues (Mooney and Kanyeredzi 2021). Arguing for a focus on nurses' needs entails supporting the development of restorative environments in acute units, consistent with the link between a positive moral climate and reduced moral stress among nurses (Lützén et al. 2010). Hutchinson (2009) underscored that such an environment is built on shared responsibility, supportive relationships, and open dialogue to repair harm. Typified in restorative circles, a structured process and safe space where nurses can gather and maximise open discussions for challenge and growth, these environments move away from punishment and blame and towards encouraging accountability and nurturing a culture of respect and collegial support.

The current study is the third in a series that examines restrictive practices in one adult inpatient mental health unit. The current retrospective before‐and‐after study was aimed at assessing the impact of restrictive practice review meetings in the unit from January 2019 to May 2021. This investigation was aimed at testing whether investing time and resources into restorative approaches with nurses could contribute tangibly to service delivery improvements, specifically changes in the rates of seclusion, physical restraint, and Code Black.

## 2 Methods

### 2.1 Setting and Context

This study was conducted in a regional acute mental health unit in New South Wales (NSW), Australia. It is a declared 30‐bed adult inpatient unit, with a limited number of high‐observation beds. It is a government‐funded facility located within a public hospital. The unit operates 24/7, with a team of nurses, a nurse educator, a clinical nurse educator, and nurse unit managers.

In this unit, a nurse unit manager (PG) envisioned how restrictive practice review meetings could directly and measurably contribute to improved service delivery. With the help of a log designed by a clinician (MF) and refined in collaboration with another clinician (AT), a rethink of restrictive practice review meetings from a deficit and clinical orientation towards a strengths‐based approach was undertaken. The log facilitated documentation of nurses' relational competencies, non‐coercive interventions, aggression levels before and after de‐escalation, physical injuries, and restrictive practice use (see Daguman et al. 2025).

From January 2019 to May 2021, in monthly review sessions lasting up to an hour, nurses engaged in reflective discussions led by the nurse unit manager employing themes aggregated from the log data on successful therapeutic responses, including acknowledgement of nurses' needs. This clinician‐led initiative aligned with broader reforms in response to the recommendations from the Review of Seclusion, Restraint and Observation of Consumers with a Mental Illness in NSW Health Facilities (NSW Health 2017) that reinforced accountability in restrictive practice use.

Prior to 2019, unit‐level reviews followed the London Protocol (Vincent 2004), which considered contributory factors, issues, and actions related to the service user, clinical management, task and technology, staff, teams, work environment, institutional context, and organisation and administrative management However, these reviews primarily focused on systemic issues and areas for improvement, without explicitly acknowledging nurses' achievements.

### 2.2 Design

The retrospective before‐and‐after design compared outcomes before and after introducing strengths‐based restrictive practice review meetings. This retrospective analysis of records is suited to examine past and rare occurrences (Gearing et al. 2006), such as restrictive practices. The before group was comprised of daily aggregates of events before the strengths‐based review meetings (16 October 2017 until 31 December 2018), while the after group referred to daily aggregates of events during the implementation of the meetings (January 2019 until 17 March 2020). Randomisation was not employed, although statistical adjustments were applied to balance covariates.

**FIGURE 1:**
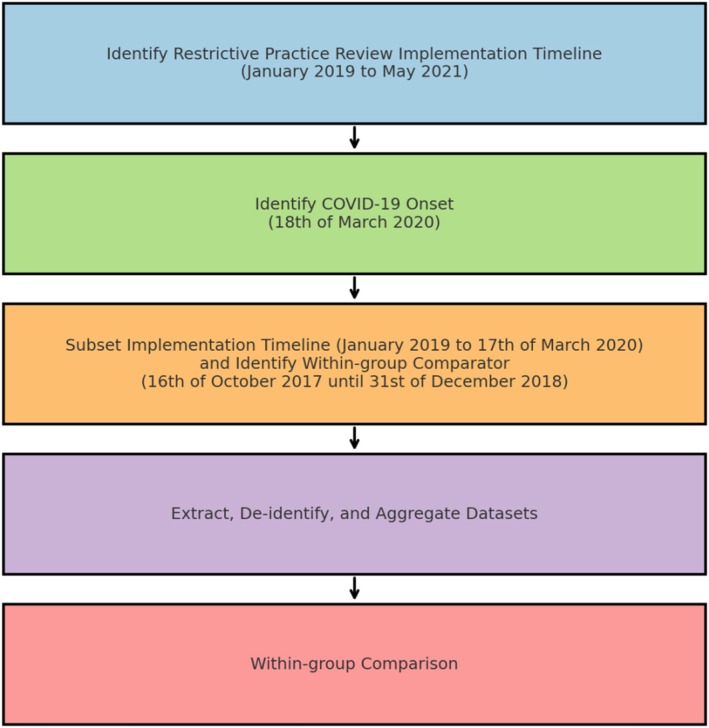
Process flow of study procedures.

An adjustment was made to separate the effects of the review meetings from those of the COVID‐19 pandemic. Following pandemic‐affected data handling guidelines (Cro et al. 2020), data influenced by the pandemic were treated as missing. The pandemic saw eased work restrictions for student nurses to assist in the pandemic response (Storen and Corrigan 2020), increasing training demands for nurse educators. Through clinical input, the identified onset of the pandemic was 18 March 2020. As a result, data from 18 March 2020 to May 2021 were set as missing. The resulting subset was compared against data from an equal period of 14 and a half months before the strengths‐based reviews (i.e., 16 October 2017 until 31 December 2018). Figure 1 outlines the process used to determine the study's observation period.

### 2.3 Procedures

Daily totals of seclusion, physical restraint, and Code Black events were considered the unit of analysis in this study, as aggregates of events offer an opportunity to assess potential reporting biases (Bowers et al. 2015). The data on seclusion and physical restraint events were from the unit's historical, legally mandated reports submitted to the National Seclusion and Restraint Database (AIHW 2024). This system gathers similar data from public hospital acute mental health units across Australia that provide short‐term care. Reports are published online to support benchmarking performance against other states, national rates, and similar services, and promote quality service delivery. Code Black events were sourced from the unit's incident management system (ims+).

All seclusion and physical restraint data undergo integrity checks. Similarly, all incidents identified and recorded through the ims+ are flagged for review and action, checked for accuracy, and responded to promptly by management. The standard reports on seclusion and physical restraint data are contemporary extensions of the ims+ reports and are cross‐referenced against each other. This cross‐referencing ensures that incidents of Code Blacks are accurately recorded and reviewed. These data checks suggest the validity and reliability of the data used in this study. Days recorded without any events were actual zero events and not missing data.

Based on the mixed‐effects model specifications described in the Data Analysis section, synthetic data were generated from daily totals to match the maximum values and distribution in the outcome data. Monte Carlo simulations in the simr package (Green et al. 2023) showed that 3 months of data are needed to detect a 12% drop in seclusion with 99.30% power, similar to the effect size in an NSW Safewards study (Dickens et al. 2020). At a similar effect size, achieving 90.6% power for physical restraint requires 10 months, while 85.20% power for Code Black requires 9 months. The NSW Safewards evaluation was chosen as a reference for power analysis due to its high methodological quality, being the only study identified in an overview of systematic reviews (Daguman et al. 2024) comparable to the current study based on pre/post‐test design and geographical context.

In order to ensure privacy, a designated staff member, authorised by the data custodian, de‐identified the data by removing all identifying information of nurses and service users involved in outcome incidents. Data were aggregated and analysed by the first author, who remained blind to the identities of reporting nurses and the service users involved, to minimise bias arising from the measurement of the outcome data.

The data custodian institution reviewed this study (Ref: QA2024_05), which used only non‐identifiable data, classifying it as low‐risk and exempting it from Human Research Ethics Committee (HREC) approval. A relevant university HREC granted ethical approval (Approval No: 2024/145) for its conduct.

### 2.4 Data Analysis

The data analysis process began with calculating propensity scores using the IPW package (van der Wal and Geskus 2011), to mirror randomisation and balance baseline characteristics. Propensity scores represent the probability of a daily aggregate of events to be in either the ‘before’ or ‘after’ group based on observed characteristics, which was the average harm score of the daily event aggregate. These scores were then used in IPW, where each observation was weighted by the inverse of its probability of group assignment. This process created a pseudo‐population in which the distribution of covariates was balanced across both periods. A harm score is a level on a scale that evaluates the impact severity of clinical incidents (NSW Health 2020). It ranges from 4 (no harm or near miss) to 1 (severe or permanent harm, including death). The use and reporting of IPW were based on a guideline that ensures the reproducibility of research (Bettega et al. 2024).

IPW was applied in negative binomial mixed effect models to estimate outcomes (seclusion, physical restraint, and Code Black), while controlling for hierarchical effects related to months of the daily aggregate of events. A negative binomial mixed‐effects model handles over‐dispersion and clustering better than the standard Poisson model for count data with extra variability (Cameron and Trivedi 2013). The choice of month as a random effect was informed by time‐related variables in evaluations of interventions to reduce containment (Bowers et al. 2015). Outcomes of the models were reported as incidence rate ratios (IRR), which represent the relative change in the rate of restrictive practices per unit change in time. An IRR > 1 indicates an increased likelihood of restrictive practice use over time, while an IRR < 1 suggests a decrease in these events. Values are interpreted in relation to the pre‐review period as the reference category.

A synthetic dataset with two time points (0 = before, 1 = after) and the mean rounded value of the month was used to forecast outcomes under conditions similar to those of the fitted negative binomial mixed effect models. With standard error as the basis for the measure of confidence, predictions were calculated on a log scale and were back‐transformed into the original scale. As shown in Figure 3, the ribbon plots present predicted values for each outcome, with confidence intervals as ribbons.

**FIGURE 2:**
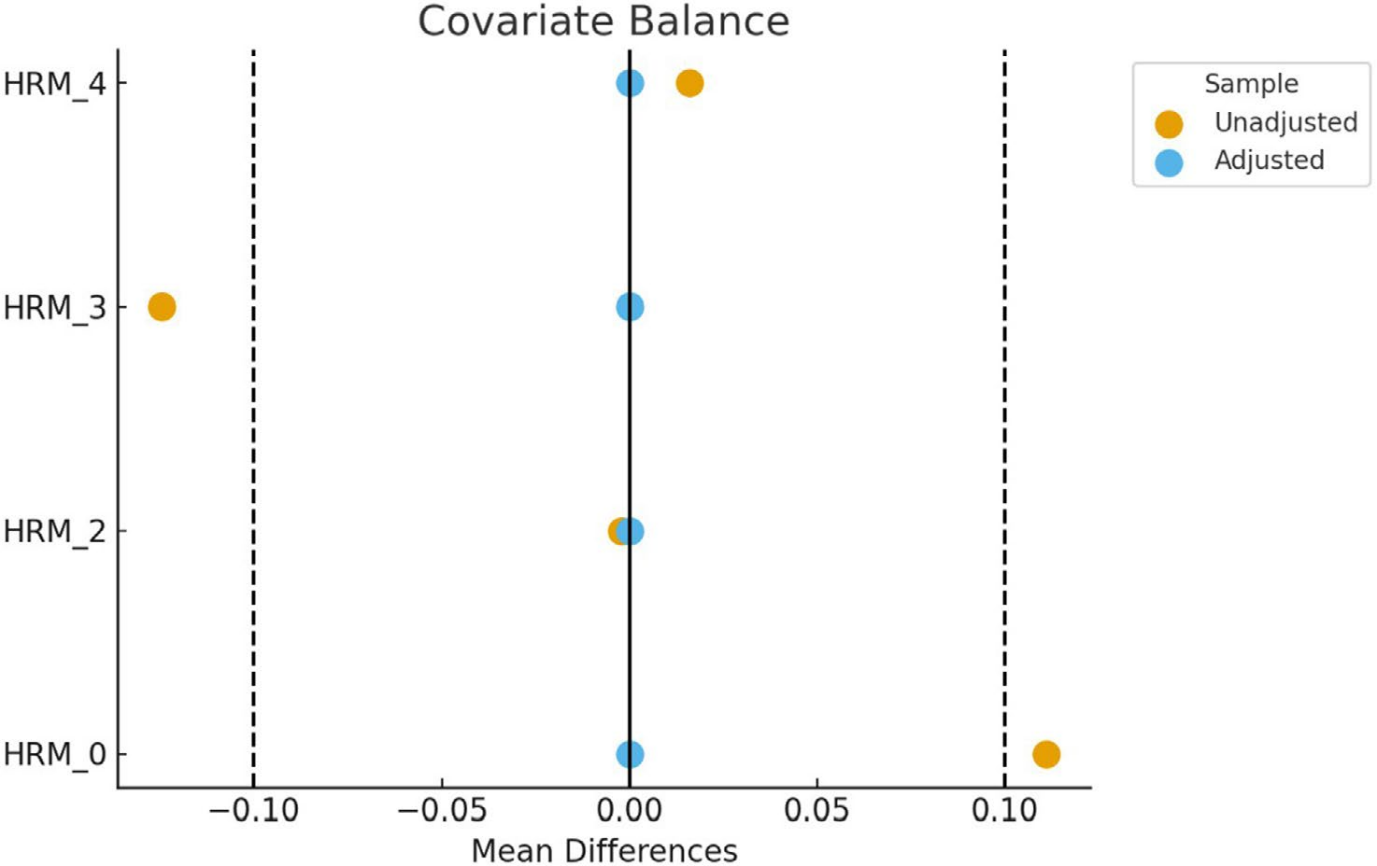
Love plot on covariate balance for harm scores of daily event aggregates.

Covariate balance was checked using a Love plot in cobalt (Greifer 2024; see Figure 2). Model diagnostics were undertaken to evaluate model fit, and explanatory power was estimated using delta coefficients of determination (Nakagawa et al. 2017). Over‐dispersion and likelihood ratio tests were used to check if a negative binomial mixed effect model should be used instead of the Poisson counterpart (Hilbe et al. 2013). All statistical analyses were undertaken in RStudio (Posit Team 2023), whereas the visualisations were plotted through Matplotlib (Hunter 2007).

## 3 Results

The diagnostic results for the negative binomial mixed‐effects models yielded very low conditional *R* ^2^ values: 0.05 for seclusion, 0.01 for physical restraint, and 0.02 for Code Blacks, suggesting very weak explanatory power. Similarly, the marginal *R* ^2^ values were very low, with 0.03 for seclusion, 0.003 for physical restraint, and 1.51 × 10^−5^ for Code Blacks. The intraclass correlation coefficients were 0.05 for the seclusion model, 0.03 for physical restraint, and 0.06 for Code Blacks, indicating that the random effect explains only a small portion of the total variance. On the other hand, the likelihood ratio tests confirmed the appropriateness of negative binomial mixed effect models, compared to their Poisson counterparts. No over‐dispersion, zero‐inflation, or singular fit was observed. The Love plot indicated that covariate balance was achieved across groups after applying IPW (see Figure 2).

Following the implementation of strengths‐based reviews, the number of seclusion and physical restraint events decreased, while Code Black slightly increased (see Table 1). Before the strengths‐based reviews, there were 83 seclusions, 99 physical restraints, and 69 Code Black incidents, with most occurring in Harm Score Level 3 cases. A single Code Black incident was recorded in Harm Score Level 2. However, no seclusion or restraint events occurred at this level in either period. After the reviews, seclusion dropped to 30, physical restraint to 80 and Code Black incidents increased to 72. Harm Score Level 4 saw a slight increase in Code Black incidents.

**TABLE 1:** Frequency of seclusion, physical restraint, and Code Black by daily average harm score levels.

	Seclusion	Physical restraint	Code Black
Before strengths‐based reviews	83	99	69
No reported incident with harm score	2	1	—
Daily average harm score level 2	—	—	1
Daily average harm score level 3	81	97	68
Daily average harm score level 4	—	1	—
After strengths‐based reviews	30	80	72
No reported incident with harm score	1	2	—
Daily average harm score level 2	—	—	—
Daily average harm score level 3	29	78	69
Daily average harm score level 4	—	—	3

Compared to the before group, which serves as the reference group with an IRR of 1, the after group had a 63% statistically significant lower rate of seclusion events (IRR = 0.37, 95% CI [0.24, 0.57], *p* < 0.001). For physical restraint events, the after group had a statistically insignificant 24% reduction (IRR = 0.76, 95% CI [0.53, 1.09], *p* = 0.14), compared to the before group. For Code Blacks, the after group showed a statistically insignificant slight increase (IRR = 1.02, 95% CI [0.70, 1.49], *p* = 0.91). The months of the daily aggregate of events were set as random effects in all three models.

**FIGURE 3:**
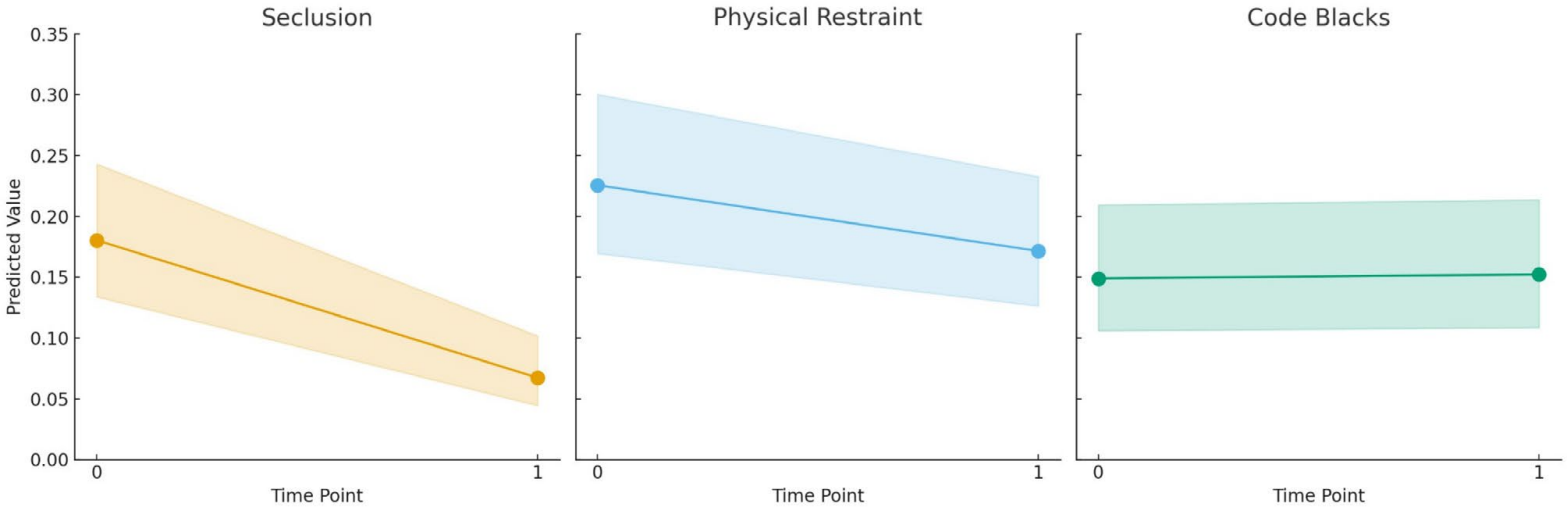
Ribbon plots of predicted rates for events of seclusion, physical restraint, and Code Blacks.

Figure 3 presents the predicted rates in a newly generated dataset for Code Black, physical restraint, and seclusion incidents, with months held at their means. Code Black incidents stay stable, with a consistent predicted mean of approximately 0.15 (95% CI [0.11, 0.21]) at both time points. In contrast, physical restraint and seclusion incidents decrease over time, with physical restraint use declining from 0.23 (95% CI [0.17, 0.30]) to 0.17 (95% CI [0.13, 0.23]), and seclusion events from 0.18 (95% CI [0.13, 0.24]) to 0.07 (95% CI [0.04, 0.10]).

## 4 Discussion

There is some support for the effectiveness of holding and receiving strengths‐based restrictive practice review meetings in reducing seclusion and restraint in this study, despite only the reduction in the seclusion outcome reaching statistical significance. These findings align well with outcomes from the work on ‘post‐seclusion and/or restraint review’ intervention, where there was an insignificant reduction in the prevalence of restraint (Goulet et al. 2018). The findings indicate that the strengths‐based reviews could have encouraged nurses' attunement to alternative strategies that stabilised service users before escalation reached the point of seclusion. The difference in statistical significance between the two outcomes suggests that seclusion is used in situations that lend themselves more readily to review and change. Given the models' coefficients of determination, it is also possible that other service‐level factors may have had a role in the differential impact seen, including levels of staffing within the unit (Price et al. 2018).

How nurses perceive physical restraint distinctly may explain this practice's insignificant reduction. Nurses see physical restraints as necessary, but also believe they should only be used as a last resort for safety (Wong and Bressington 2022). This tension may be further maintained through an overlap in perceived control (Huddy and Carey 2022), where nurses' strivings towards safety conflict with service users' efforts to work towards their goals. Nurses may have seen a situation escalating beyond verbal control and applied physical restraint. In contrast, service users may have responded to the perceived limitations to their autonomy in a way that looked troublesome for nurses, reinforcing nurses' perceived need for physical restraint. However, this explanation needs to be confirmed qualitatively within the study's context.

It may be helpful to frame the seclusion and physical restraint outcomes as reflections of their place in time and area of origin. The reduction rates predicted in this study, which are based on time rather than bed numbers, differed from the State and national rates where the unit is located. The Australian national rates for seclusion rose from seven to eight events per 1,000 bed days and physical restraint from 10 to 11 events per 1,000 bed days, both from 2017–2018 to 2019–2020 (AIHW 2024). The seclusion rate in NSW increased from six to eight events per 1,000 bed days, and the physical restraint rate climbed from eight to nine events per 1,000 bed days from 2017–2018 through 2019–2020.

What behaviour change mechanism could the reductions in seclusion and physical restraint associated with the strengths‐based reviews be attributed to? Feedback and monitoring is a behaviour change technique that includes tracking reduced restrictive practices by a nurse or other staff member and communicating them back to the unit (Baker et al. 2021). In the unit under study, this was operationalised through a log designed by a clinician (MF) and refined in collaboration with another clinician (AT). The log was used to document the relational and non‐coercive skills nurses used to de‐escalate and celebrate nurses' strengths. The themes from the log were communicated in the review meetings not only for professional development, but also to lift morale, build relationships, and deploy high standards of safe, effective care. In other words, the strengths‐based reviews aligned with the functions of, although conducted differently than, adequate clinical supervision (Proctor 1988): normative, educative, and restorative. However, this mechanism remains a key uncertainty (Skivington et al. 2024) that needs validation through qualitative research.

On the other hand, the insignificant and very slight increase in Code Black events following the introduction of strengths‐based reviews raises focal considerations, mainly as no studies have yet evaluated the influence of restorative approaches on Code Black events, nor is reducing Code Black events a key performance indicator for public acute mental health services in Australia. It prompts considerations through two central points.

The first point challenges a previous association between the presence of security guards and the increased use of physical restraint (Bowers et al. 2012). It has been said that the presence of security personnel may also enhance nurses' sense of safety during Code Black events (Wilson et al. 2024). The observed decrease in this study's physical restraint use could then suggest that Code Blacks are exchangeable protection against physical restraint, in which the availability of security guards provides an immediate buffer for nurses against applying formal coercion. Nevertheless, any display of force is unlikely to be less traumatic for service users (Muir‐Cochrane et al. 2020). It is also essential to communicate that Code Blacks place a burden on security guards, whose sense of well‐being tend to decline when they are called upon during these emergencies (Brown et al. 2022).

Considering that physical restraint might introduce a biasing path between the review meetings and Code Black events, the second point emerges: does the increase in Code Black occurrences surface more from the immediate reassurance provided by the presence of security personnel, or from a shift towards restorative approaches? There could be other unexamined factors that influenced the rise in Code Blacks. Examples include a directive within the service under study to call Code Black for any situation with potential escalation, even if it began as a de‐escalation attempt, to ensure support for containment. Previously, staff could request additional support without calling Code Black. The inverse relationship between Code Blacks and physical restraint in this study could then be considered a result of nurses' reflection that visible extra staff, called in compliance with a directive, could increase a service user's bravado, and so they may have asked security personnel to stay out of sight. However, without data on intervention timing or escalation trajectories, this remains speculative and warrants further investigation.

### 4.1 Reflecting on Reflections on Coercion

The impact of the strengths‐based review meetings is mixed. The reductions seen in seclusion and physical restraint are a cause for celebration. In contrast, the insignificance of the reduction in physical restraints and the slight increase in Code Black is a space to consolidate learning. Many services may feel sceptical about the empirical support for the review meetings at best, and resist deploying these meetings at worst. However, many things can come between providing opportunities for reflection on coercion and positive changes. Even if the impacts of the review meetings were uniformly positive, they may only have a lasting effect if leadership and service management issues are addressed (Hutchinson and Jackson 2015).

Underlying the mixed impact is a subtext on the need for transformational changes. Of course, the quality of nursing care will eventually rely upon the quality of relationships nurses maintain with service users (Hawsawi et al. 2020). However, for this to flourish, many changes need to happen, including acknowledging that hidden resistance to change may be modelled by entities bigger than strengths‐based reviews. A case in point is that, over the past 30 years, 55 public inquiries and more than 55,000 submissions have been made on mental health issues in Australia, yet many remain unheard and unresolved (Francis et al. 2022). This state of unresolved issues in mental health reflects what Stewart Clegg describes as the ‘circuits of power’ (Clegg 1989; Hutchinson et al. 2010)—the often concealed but powerful networks within systems that resist change, especially when accountability and reflection on restrictive practices challenge established norms.

Despite unheeded pleas, the idea that mixed impacts are a source of organisational growth can still be realised. Although change will need to be seen at all levels of a mental health service and beyond, other forms of improvements can still be initiated from the ground up. Levelling up how interventions are developed and evaluated can exemplify this endeavour, as other unmeasured outcomes in this study may reflect the intervention's true potential. A testament to this is the marked improvement in measures of service users' experience of the support received from the unit during 2019–2020—the same period when the strengths‐based reviews were deployed—showing a 14% increase and surpassing the 80% national benchmark (NSW Health 2021). Taking a proactive approach to developing and evaluating restorative approaches may enable services and nurses to derive optimal benefits from them.

### 4.2 Implications for Research

This exploratory study's low conditional *R* ^2^ and ICC values reflect broader issues in intervention evaluation methods, rather than being purely a limitation of this study. Although they are just a few among many metrics for evaluating a model's performance, the low values were expected, given that the study outcomes are rare (Fletcher et al. 2017) and may exhibit unusual properties that could not be fully captured by a random effect (i.e., months). The broader implication is that under‐reporting evaluation information is common in acute care reform efforts, with even basic metrics, such as confidence intervals, often not reported (see Gaynes et al. 2017). This state begs a familiar question that Hussey and Hughes (2020) raised on questionable measurement practice: could the invalidity of the findings from previous evaluations of restrictive practice reduction interventions have been hidden? Under‐reporting measurement information—or relevant evaluation metrics in this context—may weaken the validity of findings, making it difficult to confidently assess studies on mental health interventions (Daguman and Taylor 2024, 2025).

No service user involvement was included in the strengths‐based reviews. However, involving people with lived experiences of mental distress and coercion is essential. Evidence shows that some service users feel vested in sharing their experiences, which can aid restraint prevention and relationship improvement (Hammervold et al. 2022). However, others see these reviews as meaningless or tick‐boxing exercises, rather than genuine opportunities to be heard. Reviews need to be supportive and respect the service users' emotional state to be effective (Hammervold et al. 2020).

The evaluation of the strengths‐based reviews using retrospective real‐world data, unaffected by the potential influence of observation, suggests that the outcomes may demonstrate rigour in the context of genuine, everyday care provision. Future research may explore how these dynamics shift in a prospective design, where the awareness of being studied, alongside fidelity checks, could influence participation, potentially leading to resistance or bias towards socially desirable responses. At the same time, fidelity checks could support identifying what facilitates successful intervention translation into routine practice (James et al. 2017). Comparing real‐world and prospective data, while incorporating fidelity assessments, may offer a deeper understanding of how review meetings function in practice and how their outcomes differ across these contexts.

## 5 Conclusions

This study showed that regular, strengths‐based meetings to review restrictive practices can help reduce the use of seclusion and, to a certain extent, physical restraint in an adult inpatient mental health unit. Code Black incidents were stable. Nevertheless, these strengths‐based reviews may have unexpected benefits that await uncovering. Future research needs to consider robust reporting of results from evaluations of restorative approaches, including service users as review participants, and reflecting on the potential for biases in how participants and facilitators of such approaches engage and reflect on coercion.

## 6 Relevance for Clinical Practice

The findings of evaluating strengths‐based reviews in an acute unit show the importance of committing to a regular and structured process. Investing time and support into these meetings signals to nurses that their professional development and personal well‐being are valued. Reflections on coercion need to be based on restorative approaches, rather than exposing or challenging nurses' practice as inadequate. Offering and providing restorative approaches also means that facilitators of restorative circles consider maximising data on nurse strengths and paint a less biased picture of the work towards eliminating restrictive practices.

## Author Contributions

**Esario IV Daguman:** conceptualisation, methodology, software, formal analysis, investigation, resources, data curation, writing – original draft, and visualisation. **Alison Taylor:** conceptualisation, resources, and project administration. **Matthew Flowers:** conceptualisation and resources. **Richard Lakeman:** resources and writing – review and editing. **Marie Hutchinson:** conceptualisation, methodology, resources, writing – review and editing, and supervision.

## Conflicts of Interest

This research was conducted, analysed, reported, interpreted, and written independently of any involvement from the organisations funding Esario IV Daguman's PhD scholarship. The views expressed in this study are solely those of the authors and do not necessarily reflect those of the funding organisations.

## Data Availability

The datasets are not publicly available, due to conditions in the quality assurance and ethical approvals obtained.
